# Retro-orbital oedema and transient blindness following endoscopic oesophagogastroduodenoscopy: a case report

**DOI:** 10.4076/1757-1626-2-9070

**Published:** 2009-09-02

**Authors:** Tahrina Salam, Tamara Nissner, Afshin Kahin, Andrew C Coombes, Daniel G Ezra

**Affiliations:** 1 Queen Elizabeth II Hospital, Welwyn Garden Hospital9 Wexner Building, 2 Strype Street, London, N1 3LJUK; 2 Royal London HospitalsWhitechapel, E1 1BDUK; 3 St George’s HospitalTooting, LondonUK; 4 Royal London HospitalsWhitechapel, E1 1BDUK; 5 Moorfield Eye HospitalCity road, London EC1V 2PDUK

## Abstract

This case report looks at the association of an endoscopic oesophagogastroduodenoscopy and the onset of retro-orbital oedema in a young female. A literature search was performed in order to find any common factors between an endoscopic investigation and retroorbital oedema. An association between increased vascular permeability secondary to alcohol abuse and retroorbital oedema has been made. The case also describes the clinical signs of retro-orbital oedema and other possible causes. A link has been made between acute reversible retroorbital oedema following endoscopic oesophagogastroduodenoscopy.

## Case presentation

A 29 year old white female, with a history of alcohol abuse, presented to Accident & Emergency with a twelve hour history of haematemesis following an alcohol binge. On admission she was haemodynamically unstable and had evidence of recent upper gastro-intestinal (GI) blood loss. Her blood results revealed hepatic impairment with undetectable Paracetamol levels. She subsequently stabilised and an upper GI endoscopy was performed revealing haemorrhagic gastritis. It was an unremarkable procedure, performed with 5 mg of Midazolam as a sedative and no topical anaesthetic. Throughout the procedure, the patient was fully cooperative and no therapeutic procedure was needed.

Within two minutes of the endoscope being removed, the patients’ upper face and eyelids began to swell. There was no stridor at any time. Within 5 minutes, the patient complained of being completely blind. An urgent ophthalmic review documented a vision of no perception of light (NPL) in the left eye and perception of light (PL) in the right. Her pupils were both fixed and mid dilated. A severe global restriction of eye movements with severe proptosis was also noted.

An urgent CT head/orbit was performed revealing no evidence of retrobulbar haemorrhage, but marked oedema of the superficial tissues of the upper face, eyelids and retrobulbar tissues. The patient was given IV antihistamine (10 mg of chlorphenamine) and 200 mg of IV hydrocortisone, both slowly administered. The following day, the swelling and proptosis began to subside with a decrease in Hertels exophthalmometry from 21 mm to 19 mm bilaterally. Her vision improved to hand movements in both eyes. Five days later the orbital and facial swellings further improved and by the time of her discharge one week later, her vision was 6/12 bilaterally with constricted Goldman visual fields bilaterally. Now, 4 years after the event, her vision is 6/9 bilaterally with full visual fields and no concurrent medical problems.

## Discussion

This 29-year-old alcoholic female patient developed an acute reversible upper facial and retroorbital oedema. This dramatic presentation of retroorbital oedema is consistent with a localised increase in vascular permeability related to previous alcohol abuse prior to the endoscopy.

Localized retroorbital oedema without systemic symptoms has previously been reported in two patients exposed to IV contrast and patients with aspirin sensitivity [1,2]. Theses reports suggest a link with histamine release, as these patients symptoms subsided with the institution of supportive treatment and antihistamine agents [2]. The mechanism responsible for localised oedema was unclear, but in the patients with aspirin sensitivity, aspirin induced complement activation was suggested. In this study group it was noted that pre-treatment with antihistamine eliminated hypersensitivity to the drug [1]. We note that in our case, antihistamine was successfully used to treat the retroorbital oedema, suggesting that histamine release plays a role in the localised reaction. Furthermore, it is recognised that a patient *in extremis* and during a stress response will exhibit hypersensitivity reactions which are more common and pronounced [3].

Sudden onset facial swelling may have potential life threatening origins that need to be excluded. Angioedema is a common cause of facial swelling and may result from a number of different mechanisms [4]. Hereditary angioedema (HAE) is a well defined autosomal dominantly inherited defect of C1 esterase inhibitor. Our patient had no history of atopy and IgE, C1q esterase, C2, C3 and C4 levels were normal. RAST testing against common allergens, including latex, were also negative and thus a diagnosis of HAE and latex allergy were excluded. However, HAE with normal biochemical C1-inhibitor function has been reported in women [5].

Other stimuli for the onset of facial and periorbital oedema in this case have not been determined although the chronology of events in this case strongly suggests that the oedematous reaction was related to the endoscopy. Narcotics have been linked to angioedema by directly stimulating mast cell release by a non IgE mediated mechanism. The only drug used in our case report was midazolam, which may have resulted in an allergic IgE antibody mediated episode of angioedema, but this has not previously been reported [6].

**Figure 1.**
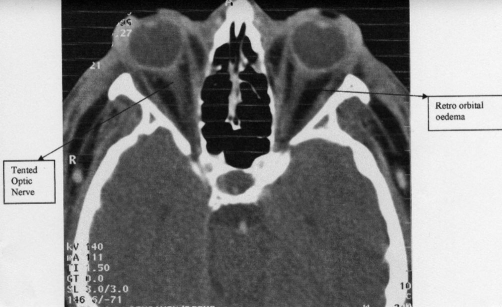
Severe oedema of retro-orbital tissues and superficial soft tissues of the face. Note the straight optic nerves and posterior tenting of the globes.

Of particular note in this report are the severity of the swelling and the dramatic nature of the symptoms. Clinical examination and imaging indicated that visual loss was due to optic nerve compromise resulting from severe proptosis (Figure 1). Yet despite almost complete blindness, her visual recovery was equally as dramatic. The rapid administration of immune modulating agents and antihistamine may have controlled the swelling early enough to limit the optic nerve damage to a reversible level. Acute optic nerve compromise with posterior globe tenting of less than 120 degrees is documented to recover with decreasing tensile stress to the nerve [7]. As this patients’ recovery was very rapid, orbital decompression was not considered. However, if symptoms and signs of optic nerve compression persisted despite conservative management, the use of decompression surgery may have been indicated. This case serves to illustrate a rare complication of oesophagogastroduodenoscopy (OGD). Ophthalmologists and gastroenterologists should be aware of this rare presentation and the importance of rapid and appropriate treatment.

## Abbreviations

C: complement
CT: computerised tomography
GI: gastrointestinal
HAE: hereditary angioedema
Ig: immunoglobulin
IV: intravenous
NPL: no perception of light
OGD: oesophagogastroduodenoscopy
PL: perception of light
RAST: radioallergosorbent test

## References

[bib-001] Katz Y, Goldberg N, Kivity S (1993). Localised periorbital oedema induced by aspirin. Allergy.

[bib-002] Sanjiv S, Upendra K, Rajani M (1990). The spectrum of isolateed ocular reactions following intravascular contrast administration. Indian Heart J.

[bib-003] Ring J, Brockow K, Behrendt H (2004). History and classification of anaphylaxis. Novartis Found Symp.

[bib-004] Orfan NA, Kolski GB (1992). Angioedema and C1 inhibitor deficiency. Ann Allergy.

[bib-005] Bork K, Banstedt SE, Koch P, Traupe H (2000). Hereditary angioedema with normal C1-inhibitor activity in women. Lancet.

[bib-006] Mastrovich JD, Peters N, Tripathi A (2004). Acute onset of facial swelling during colonoscopy in a 50 year old woman. Ann Allergy Immunol.

[bib-007] Dalley RW, Robertson WD, Rootman J (1989). Globe tenting: a sign of increased orbital tension. AJNR Am J Neuroradiol.

